# Hydrogen attenuates ischaemia–reperfusion injury in skeletal muscles post-limb replantation by activating the NRF2/HO-1 signalling pathway to reduce BAX expression

**DOI:** 10.1016/j.heliyon.2024.e37018

**Published:** 2024-09-01

**Authors:** zi-hao Jiang, jun-sheng Wang, jin-ling Wang, jiang-fan Zheng, xiao-ling Li, zhi-cheng Yang, meng-qiu Xu, yong-li Zhang, yu Wang

**Affiliations:** a Department of Emergency, Zhongshan Hospital Xiamen University, School of Medicine, Xiamen University, Xiamen, China; b Department of Emergency and Critical Care Center, The Second Affiliated Hospital of Guangdong Medical University, Zhanjiang, China

**Keywords:** Hydrogen, Ischaemia–reperfusion injury, NRF2/HO-1 pathway, Oxidative stress

## Abstract

**Background:**

Ischaemia–reperfusion injury (IRI) is a critical complication post-limb replantation. The oxidative stress and cellular apoptosis due to IRI considerably hinder the healing process. This study aimed to investigate the modulatory effects of pre-perfusion with hydrogen-rich heparin sodium on the nuclear factor erythroid 2–related factor 2 (NRF2)/haeme oxygenase-1 (HO-1) pathway and its potential mechanisms in mitigating skeletal muscle IRI post-limb replantation.

**Methods:**

Forty healthy Sprague–Dawley rats (250–300 g) were classified into five groups (n = 8 each): normal control, IRI + heparin sodium pre-perfusion (heparin group), IRI + hydrogen-rich heparin sodium pre-perfusion (hydrogen-rich heparin group), IRI + hydrogen-rich heparin sodium pre-perfusion + NRF2 inhibitor (hydrogen-rich heparin + all-trans retinoic acid [ATRA] group), and IRI + heparin sodium pre-perfusion + NRF2 inhibitor (heparin + ATRA group). The activation of the NRF2/HO-1 pathway in skeletal muscle IRI was evaluated based on HO-1 expression using western blotting and immunofluorescence. Furthermore, haematoxylin and eosin staining and transmission electron microscopy were employed to determine the histopathological characteristics. Additionally, superoxide dismutase and malondialdehyde levels in skeletal muscle tissue were measured to assess antioxidant capacity and the degree of oxidative stress damage. Tissue hypoxia was assessed based on hypoxia-inducible factor 1-alpha expression, whereas apoptosis markers BCL-2-associated X protein (BAX) and Caspase-3 in skeletal muscle tissues were analysed using western blotting with terminal deoxynucleotidyl transferase dUTP nick end labelling staining to quantify cell apoptosis.

**Results:**

Compared with the control group, the heparin group exhibited significant pathological changes, including inflammatory infiltration and cellular hypertrophy, with increased apoptosis and oxidative stress. Notably, NRF2 suppression aggravated these effects. However, hydrogen-rich heparin sodium prominently activated the NRF2/HO-1 pathway, enhancing antioxidant defence and reducing BAX/Caspase-3-mediated apoptosis, thereby mitigating IRI-induced damage. The use of an NRF2 inhibitor to inhibit NRF2 excitation by hydrogen-rich heparin sodium notably weakened NRF2 activation and the antioxidant response, resulting in a substantial increase in cellular apoptosis.

**Conclusion:**

Pre-perfusion with hydrogen-rich heparin sodium markedly diminishes the BAX/Caspase-3-mediated apoptotic pathway in skeletal muscle tissues with IRI through the excitation of the NRF2/HO-1 pathway.

## Highlights

• This study refines a model for limb reimplantation, improving blood flow obstruction and perfusion methods.
• Hydrogen-rich infusion pre-perfusion demonstrates protective effects on the skeletal muscle of amputated limbs.
• Hydrogen significantly mitigates oxidative stress injuries in the skeletal muscles of severed limbs.
• Hydrogen activates the Nrf2/HO-1 signaling pathway in skeletal muscles within a limb reimplantation model.
• The activation of the Nrf2/HO-1 pathway has an impact on Bax-mediated apoptosis.

## Graphical abstract

Mechanism underlying the molecular action of hydrogen.

**Figure:**
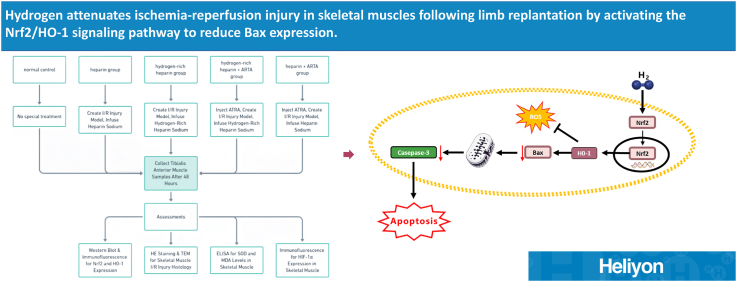

## 1 Introduction

The number of cases of multiple trauma and severe shock accompanied by limb amputation has been increasing annually. The concept of damage control is adopted to manage such cases, and an early interdisciplinary collaborative treatment, prioritising life-threatening shock and complex trauma, is implemented [1]. Notably, limb replantation is performed at an appropriate time after the vital signs of patients are stable. The optimisation of limb preservation strategies is an urgent issue for delayed limb replantation, especially in complex military environments. Compared with traditional static cold storage methods, pre-perfusion treatment of amputated limbs can effectively reduce ischaemia–reperfusion (I/R) injury (IRI) post-replantation and improve the survival rate of replanted limbs [2]. However, mature limb-perfusion solutions remain lacking in clinical practice.

Molecular hydrogen (H_2_), owing to its potential as a selective antioxidant, has garnered widespread attention in the field of medical research in recent years [3]. Hydrogen effortlessly traverses cellular barriers and directly engages in cellular processes because of its small molecular structure [4]. Its antioxidant properties are manifested in its ability to effectively neutralise harmful free radicals produced under oxidative stress, thereby mitigating cell damage [5].

Hydrogen inhalation has been shown to significantly reduce the infarct size in rat models of myocardial IRI by mitigating oxidative stress and apoptosis [6]. Additionally, clinical investigations have shown that hydrogen-rich saline improves outcomes in patients with acute myocardial infarction, as evidenced by decreased levels of oxidative stress markers and enhanced cardiac function following hydrogen therapy [7]. These findings highlight the therapeutic potential of hydrogen in alleviating IRI, indicating broader applications in oxidative stress-related conditions.

This study pioneers the investigation of hydrogen-rich perfusion solutions pre-infused into amputated limb skeletal muscles, emphasising nuclear factor erythroid 2-related factor 2 (NRF2)/haeme oxygenase-1(HO-1) pathway in IRI. NRF2 functions as a crucial intracellular regulator of the antioxidant stress response by activating various antioxidant genes [8]. HO-1, as the primary downstream target protein of NRF2, plays a crucial role in regulating oxidative stress and inflammatory responses. Stimulation of the NRF2/HO-1 pathway significantly reduces tissue damage caused by IRI and helps alleviate cellular apoptosis [9].

All-trans retinoic acid (ATRA) has been identified as a significant compound that inhibits the NRF2 pathway. The molecular structure of ATRA, a vitamin A derivative, consists of a β-ionone ring linked to a conjugated polyene chain ending in a carboxylic acid group (C_20_H_28_O_2_) [10,11]. This unique structure allows ATRA to interact with retinoic acid receptor alpha (RARα). ATRA, on binding to RARα, forms a complex that inhibits NRF2 by preventing its binding to the antioxidant response element (ARE) in the promoter regions of its target genes. This inhibition suppresses the transcriptional activation of ARE-driven genes, thus attenuating the cellular defence mechanisms against oxidative stress. Notably, NRF2 pathway inhibition by ATRA does not affect the expression or nuclear translocation of NRF2, but rather its binding ability to ARE, effectively blocking NRF2/HO-1 pathway-mediated downstream antioxidant responses [12].

Molecular hydrogen effectively alleviates I/R-induced damage in isolated rat hearts by activating NRF2 [13]. Additionally, upregulation of the NRF2/HO-1 axis can improve I/R-induced myocardial damage by reducing endoplasmic reticulum stress-related damage [14]. These findings highlight the potential role of molecular hydrogen in IRI treatment.

This study aimed to investigate how molecular hydrogen alleviates skeletal muscle damage caused by I/R by stimulating the NRF2/HO-1 pathway using a rat limb amputation model and a hydrogen-rich heparin sodium perfusion solution.

## 2 Materials and methods

### 2.1 Animals

Forty healthy male Sprague–Dawley (SD) rats weighing 250–300 g were obtained from Shanghai Shrek Experimental Animal Co. The rats were maintained in a laboratory at 25 °C, with free access to drinking water, and fed with rat growth feed. This study was approved by the Research Ethics Committee of the Animal Experiment Center at Xiamen University.

### 2.2 Main reagents and instruments

Malondialdehyde (MDA) and superoxide dismutase (SOD) testing kits were purchased from Nanjing Jiancheng Bioengineering Institute, China. Terminal deoxynucleotidyl transferase dUTP nick end labelling (TUNEL) and haematoxylin and eosin (H&E) staining kits were obtained from Servicebio, China. The antibodies used in this study are as follows: rabbit anti-rat monoclonal antibody HO-1 (Affinity, China), rabbit monoclonal antibody BAX (Cell Signaling Technology, USA), rabbit monoclonal antibody Caspase-3 (Cell Signaling Technology), mouse monoclonal antibody HIF-1α (Novusbio, USA), mouse monoclonal antibody actin (Santa Cruz, USA), horseradish peroxidase-conjugated goat anti-rabbit IgG (H + L), Alexa Fluor 488-conjugated goat anti-rabbit IgG (H + L), horseradish peroxidase-conjugated goat anti-mouse IgG (H + L), Alexa Fluor 647-conjugated goat anti-mouse IgG (H + L) (Beyotime, China). Heparin sodium injections were obtained from Shanghai Pharmaceuticals, China. The NRF2 inhibitor ATRA was obtained from Dalian Meilun, China. Hydrogen-rich water concentration determination liquid was obtained from Linyuan Technology, China. We used 9-0 fibre sutures from Ningbo Chenghe, China, and a hydrogen generator from Tuohe Electromechanical Technology Co., China. A surgical microscope (Ningbo Chenghe) and transmission electron microscope (Hitachi HT-7800, Japan) were used for microscopic observations.

### 2.3 Preparation of hydrogen-rich heparin sodium solution

The gas in a container containing 100 mL of saline solution was evacuated and replaced with high-purity hydrogen gas (purity >99.99 %). This mixture was maintained at a pressure of 0.4 MPa for 6 h [15]. The prepared solutions were stored at 4 °C under normal pressure. The concentration of hydrogen was set at saturation level (1.6 ppm) using a ‘hydrogen-rich water hydrogen concentration determination solution’. Prior to use, 12500 U of heparin sodium was added to the solution.

### 2.4 Experimental grouping and treatment

Forty male SD rats were evenly classified into five distinct groups (n = 8 each): normal control, IRI + heparin sodium pre-perfusion (heparin group), IRI + hydrogen-rich heparin sodium pre-perfusion (hydrogen-rich heparin group), IRI + hydrogen-rich heparin sodium pre-perfusion + NRF2 inhibitor (hydrogen-rich heparin + ATRA group), and IRI + heparin sodium pre-perfusion + NRF2 inhibitor (heparin + ATRA group). Except for the normal control group, all the other groups were induced limb reimplantation IRI. In the heparin group, following establishment of the IRI model, heparin sodium was perfused through the femoral artery at a rate of 2 mL/min with a total volume of 10 mL, until a clear fluid was observed in the femoral vein. The hydrogen-rich heparin group received a hydrogen-rich heparin sodium infusion following the establishment of the IRI model using the same steps as the heparin group. The hydrogen-rich heparin + ATRA and heparin + ATRA groups received intraperitoneal injections of ATRA at a dose of 7 mg/kg once daily for 2 consecutive days [12], 2 days prior to model establishment [16]. The subsequent procedures were the same as those used for the hydrogen-rich heparin and heparin groups. Anterior tibial muscle samples were collected for further experiments 48 h post-limb reattachment surgery.

### 2.5 Limb reimplantation IRI model establishment

**Fig. 1:**
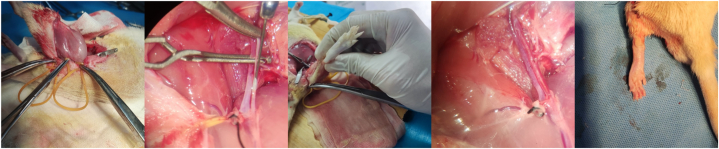
Schematic of the limb reimplantation ischaemia–reperfusion injury model establishment.

Rats were anaesthetised and disinfected routinely. An incision was made to form a ring 1 cm below the right inguinal ligament, 1.5 cm above the knee joint, and 1 cm below the pubic bone. The skin and subcutaneous tissues were cut and bluntly separated to reach the posterior part of the sartorius muscle. The sciatic nerve, femoral vein, and femoral artery were isolated and exposed [17]. The superficial epigastric arteries and veins were carefully dissected, ligated, and excised. Haemostasis of small vessel bleeding was achieved using bipolar electrocoagulation. Two non-invasive vascular clamps were used to occlude the proximal parts of the femoral vein and arteries. Tourniquet bands were applied below the fascial sheath to bind the limb, with additional bands placed around the quadriceps and hamstring muscles to interrupt collateral circulation. Under a surgical microscope, microscissors were used to make small incisions in the femoral artery and vein. A micro-irrigation needle was inserted into the femoral artery and secured with 6-0 microsutures. The respective infusion solutions were administered at an infusion rate of 2 mL/min with a total volume of 10 mL until clear fluid flowed from the femoral vein opening, indicating pallor in the toes of the rat. Next, two more noninvasive vascular clamps were used to occlude the distal parts of the femoral artery and vein. After 3.5 h, the external sheath of the blood vessels was trimmed under a surgical microscope, and the artery and vein were anastomosed tension-free with 9-0 microsutures. Clamps at the distal and proximal ends of the femoral artery and vein were released 4 h after perfusion [18]. The success of reperfusion was confirmed by good arterial pulsation, patency, and no leakage [19]. The blood vessels were kept moist using heparin saline during the entire surgical procedure. All tourniquet bands were released, and the surgical incision was sutured layer-by-layer. The distal toes showed a good blood supply, indicating successful blood perfusion. After further observation for 4 h, the reimplanted limb of the rat exhibited a rosy skin colour, sensitive capillary response, and moderate tension, confirming the successful establishment of the post-reimplantation I/R model. The anterior tibial muscle tissue was collected for subsequent experiments 48 h post-surgery (Fig. 1).

### 2.6 Analysis of indicators

#### 2.6.1 H&E staining

Anterior tibial muscle tissue samples were harvested 48 h after model establishment [20]. These samples were fixed in a 4 % formaldehyde solution for 12 h and then sectioned perpendicularly to the muscle fibres into approximately 3 × 3 × 3 mm blocks [21]. The blocks were then placed in embedding boxes, covered with gauze, and rinsed with distilled water for 12 h. Automated dehydration was performed overnight, followed by xylene clarification two times. The samples were then dipped in paraffin wax at 60 °C using a wax bath, embedded, and sliced into 4 μm sections using a paraffin slicer. The slices were processed using the warm water floating and retrieving method and dried in a slide dryer at 40 °C for 1 h. Deparaffinization, gradient alcohol hydration, staining, and covering procedures were performed following the manufacturer's instructions. Muscle cell damage, interstitial oedema, and inflammatory cell infiltration were observed under a light microscope.

#### 2.6.2 Transmission electron microscopy analysis

Tissue samples were fixed and cooled. A new scalpel blade was used to slice the tissues parallel to the muscle fibres, producing strips of 0.5 × 0.1 cm, which were then fixed at 4 °C overnight. The samples were washed thrice with phosphate-buffered saline (PBS) + sucrose buffer solution, fixed in a 1 % osmium tetroxide solution for 2 h, and washed with PBS + sucrose buffer for 15 min. Tissues were then subjected to a series of dehydration steps using ascending concentrations of ethanol and acetone [22]. Next, the samples were embedded and fixed in epoxy resin [23]. Multiple ultrathin sections were prepared using a manual rotary microtome. The sections were stained with uranyl acetate for 10 min and subsequently with lead citrate for another 10 min [24,25]. Ultrastructural changes in the skeletal muscle tissue were observed under an electron microscope at various magnifications, focusing on the organisation of myofibrils, mitochondrial structural changes, alterations in the sarcoplasmic reticulum, and infiltration of inflammatory cells.

#### 2.6.3 Immunofluorescence staining

Paraffin sections (4 μm) were cleared in xylene and serially hydrated using alcohol at different concentrations [26]. Antigen retrieval was performed using a citrate-based heating method. Sections were incubated with 3 % H_2_O_2_ and blocked with foetal bovine serum. Each slide was treated with 50 mL of diluted primary antibodies (HO-1 rabbit anti and HIF-1α mouse anti) and subjected to overnight eclosion at 4 °C. The slides were subsequently incubated with 50–100 μL of species-specific fluorescent secondary antibodies (goat anti-rabbit green and goat anti-mouse red) at room temperature for 60 min, followed by a 5-min incubation with 50–100 μL of 4′,6-diamidino-2-phenylindole (DAPI) staining solution. Finally, slides were sealed using glycerol. Notably, throughout this process, the slides were washed multiple times with PBS to maintain moisture. Fluorescent confocal microscopy was used for examination and documentation.

#### 2.6.4 TUNEL assay

The processes of embedding, sectioning, and dewaxing of the anterior tibial muscle tissues for the TUNEL assay were the same as those used for H&E staining. The assay was performed using a TUNEL Kit (Servicebio) following the manufacturer's instructions.

#### 2.6.5 Western blotting

Fresh samples of anterior tibial muscle tissues were obtained and preserved at ˗80 °C. Protein extraction was performed using tissue lysis buffer, and protein concentrations were assessed using the BCA assay [27]. Proteins were separated on 15 % SDS–PAGE at 120 V for 40 min, which were then transferred onto PVDF membranes at 300 mA for 45 min [28]. The membranes were then blocked with 5 % non-fat milk for 2 h. The membranes were then incubated overnight at 4 °C with the following primary antibodies diluted in PBS: rabbit anti-rat BAX (1:4000, Affinity), rabbit anti-rat Caspase-3 (1:3000, Affinity), rabbit anti-rat HO-1 (1:2000, Affinity), and mouse anti-rat β-actin (1:5000, Santa Cruz). After five washes with 1 × TBST buffer, the membranes were then incubated with horseradish peroxidase-conjugated secondary antibodies (1:3000, Beyotime) for 1 h at ambient temperature. The membranes were developed using ECL in a dark room, followed by exposure. Band intensities were quantitatively analysed with ImageJ software and normalised with that of β-actin.

#### 2.6.6 SOD and MDA detection

Fresh anterior tibial muscle tissues were harvested and stored at ˗80 °C. SOD activity and MDA levels in the tissues 48 h after surgery were assessed using SOD (WST-1 method) and MDA (TBA method) assay kits (Nanjing Jiancheng Bioengineering Institute), following the manufacturer's instructions.

### 2.7 Statistical analysis

The normal distribution of the experimental data for each group was assessed using the Shapiro–Wilk test. t-tests were used to analyse the data that demonstrated a normal distribution and variance homogeneity. Normally distributed quantitative data were presented as the mean ± standard deviation (SD), and SPSS software (version 26.0) was used for statistical analysis. Comparisons between groups were conducted using one-way ANOVA, employing the LSD-t method for multiple mean comparisons, with a significance threshold set at P < 0.05.

## 3 Results

### 3.1 Hydrogen-enhanced HO-1 expression in IRI post-limb reimplantation is inhibited by ATRA

**Fig. 2:**
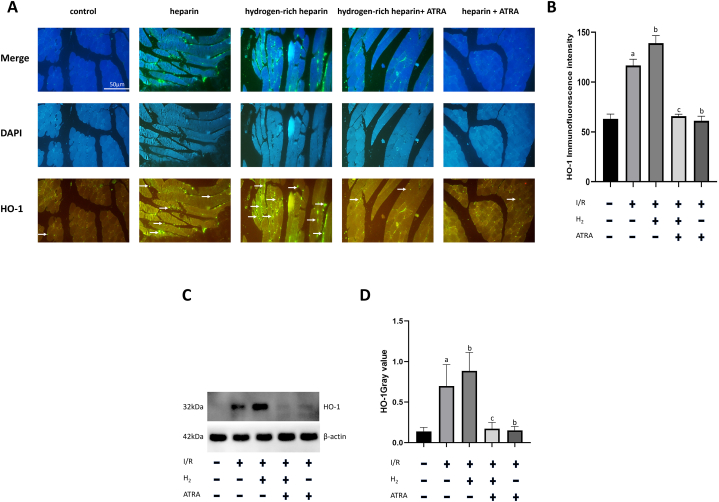
Effect of H2 on HO-1 expression in IRI and IRI + ATRA rats. (A) Immunofluorescence imaging (scale bar: 50 μm; HO-1 exposure time: 0.5 s; DAPI exposure time: 0.1 s). (B) Quantitative analysis of immunofluorescence intensity. (C) Western blotting for detecting HO-1 expression. (D) Quantitative greyscale value analysis of HO-1 expression. Data are presented as the mean ± standard deviation (SD) (n = 8). Statistical analysis was performed using one-way ANOVA with the LSD-t method for multiple comparisons. compared with the normal control group, *P*a <0.05; compared with the heparin group, *P*b < 0.05; compared with the hydrogen-rich heparin group, *P*c < 0.05. Abbreviations: IRI, ischaemia–reperfusion injury; ATRA, all-trans retinoic acid; HO-1, haeme oxygenase-1; DAPI, 4′,6-diamidino-2-phenylindole; H_2_, hydrogen.

HO-1 expression was determined based on immunofluorescence. Compared with the normal control group, the heparin group exhibited a significant increase in HO-1 expression (P < 0.05). Similarly, the hydrogen-rich heparin group exhibited a significant increase in HO-1 expression compared with that in the normal control group (P < 0.05). Furthermore, the hydrogen-rich heparin group exhibited higher HO-1 expression than the heparin group (P < 0.05). Following ATRA application, the heparin + ATRA group exhibited a significant decrease in HO-1 expression compared with that in the heparin group (P < 0.05). Similarly, the hydrogen-rich heparin + ATRA group exhibited a significant decrease in HO-1 expression compared with that in the hydrogen-rich heparin group (P < 0.05). No significant difference was observed in HO-1 expression between the heparin + ATRA and hydrogen-rich heparin + ATRA groups. Subsequently, we conducted western blot analysis to examine HO-1 expression, which yielded trends consistent with the immunofluorescence results (Fig. 2).

### 3.2 Hydrogen-rich heparin sodium pre-perfusion inhibits skeletal muscle tissue oedema, muscle fibre lysis necrosis, mitochondrial damage, and sarcoplasmic reticulum stress

**Fig. 3:**
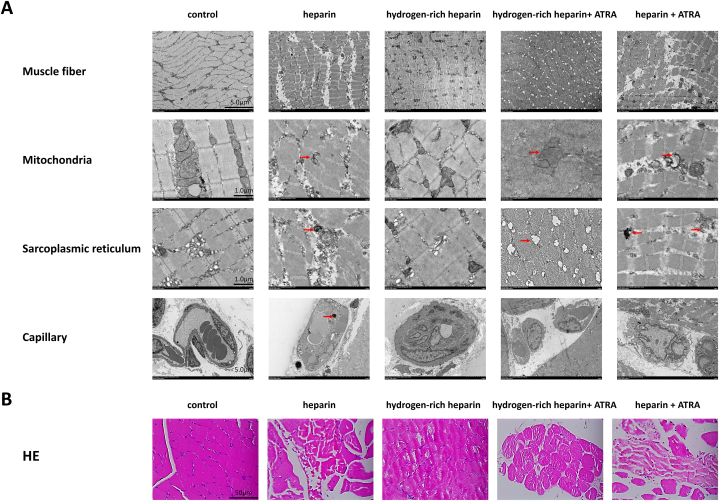
Effect of hydrogen-rich heparin sodium pre-perfusion on the cellular morphology of rat skeletal muscle post-limb reimplantation and IRI. (A) Ultrastructural view using transmission electron microscopy (scale bars: 5 and 1 μm. (B) H&E staining (scale bar: 50 μm). Abbreviations: IRI, ischaemia–reperfusion injury; H&E, haematoxylin and eosin; ATRA, all-trans retinoic acid.

The cytomorphological effects of hydrogen-rich heparin sodium pre-perfusion on skeletal muscles post-limb reimplantation and IRI in rats were assessed using transmission electron microscopy and H&E staining. In the normal control group, muscle fibres were orderly and uniform without any signs of congestion, oedema, or inflammatory infiltration; nuclei were normal with intact membranes and evenly distributed chromatin; mitochondria and the sarcoplasmic reticulum were well preserved; and glycogen in the interstitium was abundant. Conversely, the heparin group exhibited prominent myofilament disruption and disarray; nuclei were misaligned with irregular membranes and varied shapes; mitochondria with swelling, reduced cristae, and dense matrix; sarcoplasmic reticulum with scattered vesicles, indicating abnormal protein folding; significant neutrophil infiltration, erythrocyte extravasation, interstitial oedema, and glycogen depletion; and irregular blood vessels with visible necrosis, indicative of irreversible skeletal muscle damage. The hydrogen-rich heparin group exhibited minor filament disorganisation, slight myofibril swelling, and discernible Z-lines; mostly normal nuclear structures; mitochondria with minor swelling; predominantly normal sarcoplasmic reticulum structures with few vacuoles; mild neutrophil infiltration, slight interstitial oedema, and a minor decrease in glycogen; and normal vascular structures with no notable necrosis. Notably, the protective effect of hydrogen diminished after ATRA application. The hydrogen-rich heparin + ATRA group exhibited marked myofibril contraction and disorganisation; significant nuclear shape variation with irregular membranes and chromatin clumping; severe mitochondrial swelling, significantly reduced cristae and dense matrix; dispersed sarcoplasmic reticulum with dilated tubules; and extensive neutrophil infiltration, interstitial oedema, glycogen depletion, and necrosis. The heparin + ATRA group exhibited even more drastic changes, including pronounced myofilament disorder, varied sizes, and widened spaces; extreme nuclear shape variation and disorganisation, with some nuclei rupturing; mitochondrial structure collapse; dispersed sarcoplasmic reticulum forming numerous vesicles, indicative of abnormal protein folding; significant neutrophil and erythrocyte infiltration; marked interstitial oedema, glycogen depletion, and extensive necrotic areas; and ruptured blood vessels, signifying severe skeletal muscle inflammation and damage (Fig. 3).

### 3.3 Hydrogen-rich heparin sodium pre-perfusion relieves the hypoxic state in rat skeletal muscle tissues post-limb reimplantation and IRI, but is ineffective in rats with IRI following ATRA application

**Fig. 4:**
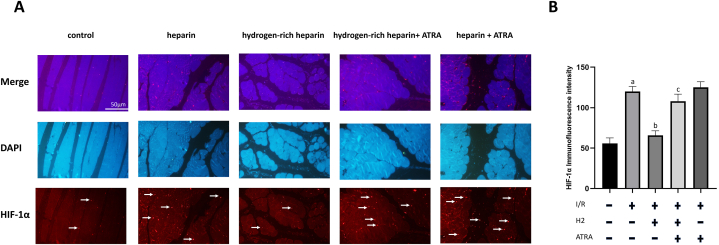
Effect of H2 on HIF-1α expression in rats with IRI and IRI + ATRA. (A) Immunofluorescence (scale bar: 50 μm; HIF-1α exposure time: 0.5 s; DAPI exposure time: 0.1 s). (B) Quantitative analysis of immunofluorescence intensity. Data are presented as the mean ± standard deviation (SD) (n = 8). Statistical analysis was performed using one-way ANOVA with the LSD-t method for multiple comparisons. Compared with the normal control group, *P*a <0.05; compared with the heparin group, *P*b < 0.05; compared with the hydrogen-rich heparin group, *P*c < 0.05. Abbreviations: IRI, ischaemia–reperfusion injury; ATRA, all-trans retinoic acid; HIF-1α, hypoxia-inducible factor 1-alpha; DAPI, 4′,6-diamidino-2-phenylindole; H_2_, hydrogen.

The effect of hydrogen-rich heparin sodium infusion on the hypoxic state of skeletal muscle tissues was analysed based on HIF-1α expression using immunofluorescence. Compared with the normal control group, the heparin group exhibited a significant increase in HIF-1α expression (P < 0.05). Conversely, compared with the heparin group, the hydrogen-rich heparin group exhibited a significant decrease in HIF-1α expression (P < 0.05). Following ATRA application, the heparin + ATRA group exhibited a significant increase in HIF-1α expression compared with that in the normal control group (P < 0.05). Similarly, the hydrogen-rich heparin + ATRA group exhibited a significant increase in HIF-1α expression compared with that in the normal control group (P < 0.05). Compared with the hydrogen-rich heparin group, the hydrogen-rich heparin + ATRA group exhibited a significant increase in HIF-1α expression (P < 0.05). No significant difference was observed in HIF-1α expression between the heparin + ATRA and hydrogen-rich heparin + ATRA groups (Fig. 4).

### 3.4 Hydrogen-rich heparin sodium infusion reverses the imbalance of oxidative stress status in rats post-limb reimplantation and IRI, but is ineffective in ATRA-treated rats

**Fig. 5:**
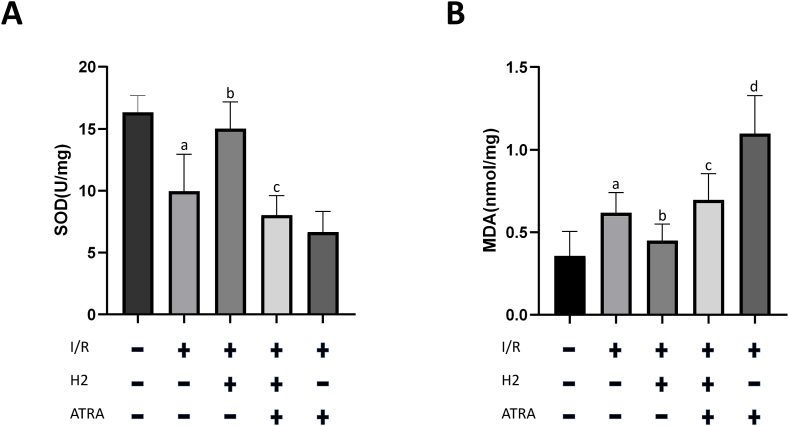
Effect of H2 on SOD enzyme activity and oxidative products in rat skeletal muscles post-limb reimplantation and IRI. Anterior tibial muscles were assessed 48 h post-limb reimplantation surgery. (A) SOD activity. (B) MDA level. Data are presented as the mean ± standard deviation (SD) (n = 8). Statistical analysis was performed using one-way ANOVA with the LSD-t method for multiple comparisons. Compared with the normal control group, *P*a <0.05; compared with the heparin group, *P*b < 0.05; compared with the hydrogen-rich heparin group, *P*c < 0.05; compared with the hydrogen-rich heparin + ATRA group, *P*_*d*_ < 0.05. Abbreviations: IRI, ischaemia–reperfusion injury; SOD, superoxide dismutase; MDA, malondialdehyde; ATRA, all-trans retinoic acid; H2, hydrogen.

Compared with the normal control group, the heparin group exhibited a significant decrease in SOD activity and increase in the oxidative product, MDA (P < 0.05). Hydrogen-rich heparin sodium infusion significantly increased SOD activity and decreased MDA levels in rats post-limb reimplantation and IRI (P < 0.05). Following ATRA application, the heparin + ATRA group exhibited a significant increase in MDA levels compared with that in the heparin group (P < 0.05). Similarly, the hydrogen-rich heparin + ATRA group exhibited a significant increase in MDA levels compared with that in the hydrogen-rich heparin group (P < 0.05). However, compared with the heparin + ATRA group, the hydrogen-rich heparin + ATRA group exhibited a significant decrease in MDA levels (P < 0.05), which may be related to the ability of hydrogen to directly neutralise some reactive oxygen species (ROS) (Fig. 5).

### 3.5 Hydrogen-rich heparin sodium perfusion solution reduces BAX/Caspase-3-mediated apoptosis in the skeletal muscle of rats with IRI post-replantation of amputated limb, but is ineffective in ATRA-treated rats

**Fig. 6:**
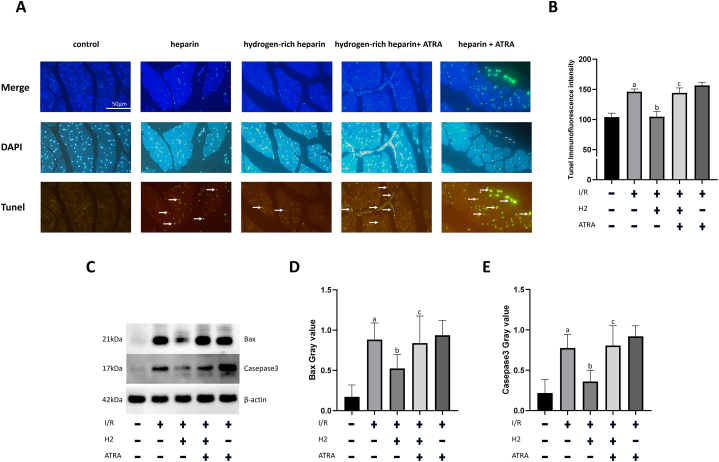
Effect of H2 on apoptosis in rat skeletal muscle tissue following ischaemia–reperfusion injury post-limb replantation. (A) TUNEL assay (scale bar: 50 μm; TUNEL exposure time: 0.6 s; DAPI exposure time: 0.1 s). (B) Quantitative analysis of TUNEL fluorescence intensity. (C) Western blotting to detect BAX and Caspase-3 expression. (D) Quantitative analysis of the greyscale value of BAX expression. (E) Quantitative analysis of the greyscale value of Caspase-3 expression. Data are presented as the mean ± standard deviation (SD) (n = 8). Statistical analysis was performed using one-way ANOVA with the LSD-t method for multiple comparisons. Compared with the normal control group, *P*a <0.05; compared with the heparin group, *P*b < 0.05; compared with the hydrogen-rich heparin group, *P*c < 0.05. Abbreviations: IRI, ischaemia–reperfusion injury; TUNEL, terminal deoxynucleotidyl transferase dUTP nick end labelling; DAPI, 4′,6-diamidino-2-phenylindole; BAX, BCL-2-associated X protein; H_2_, hydrogen.

Compared with the normal control group, a notable elevation in TUNEL fluorescence intensity was observed in the heparin group (P < 0.05). Compared with the heparin group, the hydrogen-rich heparin group exhibited a decrease in TUNEL fluorescence intensity (P < 0.05). Following ATRA application, the hydrogen-rich heparin + ATRA group exhibited a higher TUNEL fluorescence intensity than the hydrogen-rich heparin group (P < 0.05). No significant difference was observed in TUNEL fluorescence intensity between the hydrogen-rich heparin + ATRA and heparin + ATRA groups (Fig. 6A and B). Notably, prominent differences in mitochondrial changes were observed among the groups under a transmission electron microscope, prompting the use of western blotting to detect the mitochondrial apoptotic pathway components BAX and Caspase-3. Compared with the control group, the rat skeletal muscle tissue in the heparin group exhibited increased BAX and Caspase-3 expression (P < 0.05). Conversely, the hydrogen-rich heparin group exhibited decreased BAX and Caspase-3 expression compared with that in the heparin group (P < 0.05). The NRF2 inhibitor group showed further increases in BAX and Caspase-3 expression in rat skeletal muscle tissues. Compared with the hydrogen-rich heparin group, the hydrogen-rich heparin + ATRA group exhibited increased BAX and Caspase-3 expression (P < 0.05) (Fig. 6C, D, E).

## 4 Discussion

IRI, a prevalent and critical complication of limb replantation surgery, significantly affects the survival of the replanted limb and the postsurgical recovery process. IRI involves complex pathophysiological mechanisms and is characterised by a substantial increase in tissue cellular oxidative stress and exacerbated cellular apoptosis. These changes pose a serious challenge to post-surgical recovery and the maintenance of organ function. At the cellular level, IRI primarily involves mitochondrial dysfunction, activation of cellular autophagy, activation of apoptotic pathways, and imbalanced release of various effector molecules [29,30]. Notably, the core mechanism underlies the release of damage-associated molecular patterns and the activation of pattern recognition receptors [31], which subsequently activate the nuclear factor kappa-light-chain-enhancer of activated B cells. Additionally, increased NADPH oxidase activity in the mitochondria results in the production of a large amount of ROS [32]. These changes ultimately damage cells and affect tissue repair and functional recovery. Therefore, alleviating IRI, especially in the skeletal muscle tissues, is crucial for improving the success rate of limb replantation surgeries.

Several studies have emphasised the role of oxidative stress in IRI. ROS play a crucial role in mediating cellular injury during IRI, primarily through lipid peroxidation, protein oxidation, and DNA damage. Strategies for mitigating ROS production can protect the tissues from IRI. Antioxidants such as N-acetylcysteine significantly reduced oxidative stress and protected cardiac tissue in a rat model of myocardial IRI [33], which is also consistent with our finding that hydrogen can reduce oxidative stress in skeletal muscles during limb replantation.

During IRI, HIF-1α plays a dual role in the cellular response to hypoxia, as it aids in cellular adaptation during the hypoxic response and can exacerbate inflammation and oxidative stress, thereby increasing tissue damage. HIF-1α expression increases with the severity of hypoxia [34], and HIF-1α in hypoxic conditions regulates the expression of a series of genes, such as *Vegf*, which are involved in various biological processes, including angiogenesis and cellular metabolic regulation, to adapt to the hypoxic environment [35]. However, in IRI, HIF-1α activation may cause negative effects, such as promoting inflammatory responses and increasing vascular permeability, thereby exacerbating tissue damage [36]. Moreover, HIF-1α activation exacerbates oxidative stress by amplifying ROS production, which can then stabilise HIF-1α, resulting in additional tissue damage [37].

Our findings revealed that hydrogen-rich heparin sodium perfusion significantly reduced HIF-1α expression, which is a key regulatory factor in the cellular response to hypoxic environments. Notably, the reduction in HIF-1α expression indicates an improved tissue oxygenation state, consistent with findings from studies where modulating HIF-1α expression influenced IRI outcomes. For example, inhibiting HIF-1α expression in a mouse model of renal IRI reduced inflammation and tissue damage [38]. Therefore, hydrogen may exert its protective effects through HIF-1α.

In contrast to the HIF-1α role, NRF2 and its downstream target HO-1 exhibit a protective role in IRI. Under normal conditions, NRF2 remains inactive in the cytoplasm and binds to its inhibitor, KEAP1. However, under oxidative stress, NRF2 detaches from KEAP1 and translocates to the nucleus, subsequently enhancing the expression of several antioxidant genes, such as that encoding HO-1 [39]. HO-1 catalyses haeme breakdown to produce carbon monoxide, biliverdin, and free iron ions, all of which exhibit antioxidant, anti-inflammatory, and cell-protective properties, effectively alleviating the tissue damage caused by I/R [40].

In 2007, Professor Ikuroh Ohsawa proposed that hydrogen molecules can selectively scavenge hydroxyl radicals, thereby treating oxidative stress [3]. Notably, hydrogen gas can alleviate the damage caused by oxidative stress by affecting the NRF2 pathway [41,42]. Hydrogen gas affects oxidative stress and exerts anti-inflammatory and anti-apoptotic effects in various organs, including the brain, heart, pancreas, lungs, and liver [43,44]. In addition, hydrogen is significantly advantageous for preserving organs ex vivo, and organ preservation solutions containing hydrogen gas are more advantageous than those without hydrogen for preserving kidneys ex vivo [45].

One such preservation solution is the histidine–tryptophan–ketoglutarate (HTK) solution, which is commonly used in clinical settings for organ preservation because of its efficacy in reducing cellular metabolism and maintaining organ viability during cold storage [46]. HTK solution creates a low-viscosity environment that protects cells from ischaemic injury [47]. HTK preservation solutions with added hydrogen gas further enhance the protective effect by inhibiting cold ischaemia-induced oxidative stress reactions, suppressing the upregulation of inflammatory mediators, and reducing cell apoptosis. This combination is particularly effective for improving the preservation of heart transplants under prolonged cold ischaemia [48].

As cardiac and skeletal muscles are both striated and have similar structures and functions, these findings may be applicable to skeletal muscle preservation. Therefore, we speculated that pre-perfusion with hydrogen-rich heparin sodium in amputated limbs may provide a protective effect on skeletal muscles, similar to the benefits observed in cardiac muscle preservation with hydrogen-enhanced HTK solution.

We established a limb replantation model to investigate whether hydrogen alleviates IRI post-limb replantation via the NRF2 pathway. In early attempts, a limb replantation model was constructed by severing all muscles and bones in the lower limbs of rats. However, this method resulted in a high rate of limb necrosis and low postoperative survival in rats. Moreover, constructing the model by merely using tension bands or vascular clamps to block blood flow, although relatively mild, cannot adequately simulate limb severance, mainly because of the collateral circulation, which may affect the accuracy of the model evaluation. Consequently, we aimed to improve the limb replantation model. Notably, we simultaneously used multiple tension bands to effectively block the blood flow. The bands encircled the anterior and posterior muscle groups of the rat femur and directly surrounded the femur, effectively blocking collateral circulation. Subsequently, perfusion was performed via femoral artery cannulation, and the footpads of rats remained pale within 4 h of vessel reperfusion, confirming effective blood flow obstruction. Therefore, we successfully established a more reliable limb replantation model, avoiding the high risk associated with complete severance.

Subsequently, an NRF2 blockade model was established using the NRF2 inhibitor ATRA. Notably, ATRA competitively binds with NRF2 and prevents its interaction with ARE without affecting its expression [12]. We assessed the blockade effect by measuring HO-1 expression, the key target of NRF2. In the I/R model, HO-1 expression in the hydrogen-treated group was significantly higher than that in the normal perfusion and control groups, indicating that hydrogen activated the NRF2/HO-1 pathway. However, HO-1 expression significantly decreased following ATRA treatment even in a hydrogen-rich environment, confirming the successful construction of the blockade model. Furthermore, we used transmission electron microscopy and H&E staining to evaluate the effects of hydrogen solution on the morphological characteristics of cells after IRI. The hydrogen-rich treatment significantly improved cellular morphology by reducing myofibril fragmentation, maintaining the integrity of the nuclei and mitochondrial structures, and alleviating endoplasmic reticulum stress responses. However, these changes were not apparent in the ATRA-treated groups. Moreover, the hydrogen-rich heparin sodium perfusion significantly reduced HIF-1α expression, which is a key regulatory factor in the cellular response to hypoxic environment. The reduction in HIF-1α expression indicated an improved tissue oxygenation state. Additionally, hydrogen treatment increased SOD activity and reduced MDA levels. SOD, a key antioxidant enzyme, plays a vital role in neutralising harmful superoxide radicals, whereas the reduction in MDA levels, the final product of lipid peroxidation, indicates the protection of cell membrane integrity [49]. However, these protective effects were limited in the ATRA-treated group, further highlighting the central role of the NRF2 pathway in regulating the protective effects of hydrogen. However, in the absence of NRF2 regulation, cells could not effectively resist the damage caused by I/R, even in a hydrogen-rich environment.

Transmission electron microscopy revealed significant differences in mitochondrial morphology and structure among the experimental groups. The structural integrity of mitochondria plays a decisive role in maintaining cellular energy metabolism, alleviating oxidative stress, and reducing the rate of cellular apoptosis. Consequently, we hypothesised that hydrogen-rich heparin sodium solution could reduce cell apoptosis caused by mitochondrial pathways by stimulating the NRF2/HO-1 signalling pathway. To test this hypothesis, we performed TUNEL staining, which revealed that the hydrogen-rich heparin sodium solution significantly reduced the rate of cell apoptosis in the IRI model. However, the protective effect of hydrogen was reversed following the ATRA application. To further explore the mitochondrial dependence of apoptosis, we assessed BAX and Caspase-3 expression using western blotting. In the IRI model, hydrogen treatment significantly reduced BAX and Caspase-3 expression, indicating a substantial inhibitory effect on the apoptotic pathway. However, this effect was not observed in the ATRA-treated mouse model. In summary, in IRI post-limb replantation, hydrogen gas effectively reduced cell apoptosis caused by mitochondrial pathways by activating the NRF2/HO-1 signalling pathway, thereby significantly alleviating skeletal muscle IRI. Furthermore, the protective effect of hydrogen was significantly weakened by inhibiting NRF2 signalling, confirming the key role of the NRF2/HO-1 pathway underlying the protective effects of hydrogen.

## 5 Conclusion

Activation of the NRF2/HO-1 pathway by hydrogen significantly reduces apoptosis in skeletal muscle cells through BAX/Caspase-3, offering substantial protection against IRI post-limb replantation.

## Data availability statement

Data included in article/supplementary material/referenced in article.

## Funding

This work was supported by the fund of the Critical and Severe Sub-specialty of the Emergency Department of 10.13039/501100010108Zhongshan Hospital 10.13039/501100008865Xiamen University (No.050150).

## Ethics committee approval

This study was reviewed and approved by [the Research Ethics Committee of the Animal Experiment Center at Xiamen University] with the approval number: [XMULAC20230027], dated [April 2022].

## CRediT authorship contribution statement

**zi-hao Jiang:** Writing – review & editing, Writing – original draft, Visualization, Validation, Supervision, Software, Resources, Project administration, Methodology, Investigation, Funding acquisition, Formal analysis, Data curation, Conceptualization. **jun-sheng Wang:** Visualization, Validation, Supervision, Software, Resources, Project administration, Methodology, Investigation, Formal analysis, Data curation, Conceptualization. **jin-ling Wang:** Resources, Software. **jiang-fan Zheng:** Visualization, Validation, Data curation, Conceptualization. **xiao-ling Li:** Visualization, Validation, Resources, Project administration. **zhi-cheng Yang:** Visualization, Investigation, Formal analysis, Data curation. **meng-qiu Xu:** Formal analysis, Data curation. **yong-li Zhang:** Visualization, Validation, Supervision, Project administration, Methodology, Investigation, Funding acquisition, Conceptualization. **yu Wang:** Validation, Supervision, Software, Resources, Investigation, Funding acquisition, Conceptualization.

## Declaration of competing interest

The authors declare that they have no known competing financial interests or personal relationships that could have appeared to influence the work reported in this paper.
